# Franco-American Medical Ethics

**Published:** 1914-04

**Authors:** 


FRANCO-AMERICAN MEDICAL ETHICS. The *Jour. de Med. de Bordeaux,* August 17, 1913, reprints the two following advertisements from a daily paper: American millionaire, coming express to Bordeaux, would appreciate recommendation of physicians competent to cure his tuberculous daughter and syphilitic son. One hundred thousand francs reward if cured. Address ...... American Millionaire thanks his correspondents and selects Dr. Tancrede of Bordeaux, almost all the letters received recommending him for his tuberculous daughter and syphilitic son.

